# Design of Pulse Waveform for Waveform Division Multiple Access UWB Wireless Communication System

**DOI:** 10.1155/2014/171875

**Published:** 2014-02-11

**Authors:** Zhendong Yin, Zhirui Wang, Xiaohui Liu, Zhilu Wu

**Affiliations:** School of Electronics and Information Engineering, Harbin Institute of Technology, Harbin 150001, China

## Abstract

A new multiple access scheme, Waveform Division Multiple Access (WDMA) based on the orthogonal wavelet function, is presented. After studying the correlation properties of different categories of single wavelet functions, the one with the best correlation property will be chosen as the foundation for combined waveform. In the communication system, each user is assigned to different combined orthogonal waveform. Demonstrated by simulation, combined waveform is more suitable than single wavelet function to be a communication medium in WDMA system. Due to the excellent orthogonality, the bit error rate (BER) of multiuser with combined waveforms is so close to that of single user in a synchronous system. That is to say, the multiple access interference (MAI) is almost eliminated. Furthermore, even in an asynchronous system without multiuser detection after matched filters, the result is still pretty ideal and satisfactory by using the third combination mode that will be mentioned in the study.

## 1. Introduction

There are two definitions of UWB system: the bandwidth of signal spectrum exceeds 25% of the center frequency or the −10 dB bandwidth of signal is over 500 MHz [1]. The notion of ultrawideband (UWB) was presented in the 1990s. Due to its admirable characteristics, it has attracted attention of researchers in many fields. Such as high data transmission rate [2], strong multi-path resolution [3], high interference resistance [4], low power density [5], and so on.

In recent years, multiple access UWB communication has been a hot research topic as the development of UWB technologies. In UWB communication systems, the multiple access approaches are commonly divided into two paths: time hopping (TH-UWB) and direct sequence (DS-UWB) [6, 7]. In a sense, the multiple access scheme of DS-UWB is similar to code division multiple access (CDMA) systems; both of them use PN codes to distinguish different users; DS-UWB systems also suffer from the multiple access interference [8–10]. So the communication rate and spectrum utilization have been limited. Therefore, improving the communication rate has become a focus of research.

Among all the techniques in WDMA-UWB communications systems, it is of great importance to choose the analog waveform to denote a message symbol. In UWB systems, Rayleigh pulse and Gaussian monocycle [11] are generally used as the waveforms. In [12, 13], it presents a flexible and systematic method for generating UWB pulses that have many advantages over the Gaussian monocycle pulse. In that mechanism, all the waveforms of different users are the same, resulting in severe multiple access interference. Therefore, the utilization of orthogonal pulse shape is put forward to reduce the effects of MAI by allocating each user with a different waveform. In recent years, some orthogonal functions have been utilized in the corresponding theoretical investigations. The functions often used are modified Hermite functions [14, 15], prolate spheroidal wave functions [16], and the combination of wavelet functions and scaling functions [2]. Additionally, Wang et al. [17] presented a waveform design approach based on B-splines to obtain orthogonal waveforms which can be put into use in UWB systems.

In this study, orthogonal wavelet is another example that can satisfy the requirement of orthogonality [18], and its orthogonality includes two aspects: translation orthogonality and duration orthogonality. The first aspect, translation orthogonality, means that a waveform itself and a series of waveforms translated by given time parameters are mutually orthogonal. This property is suitable for a synchronous system, but it will cause high MAI in an asynchronous system, as at any time, the orthogonality cannot be guaranteed. The second aspect, the original waveform and the dilation/contraction waveforms are mutually orthogonal, which is called duration orthogonality. However, it is possibly not proper for UWB systems for the reason that the spectrum of these waveforms occupies different frequency bands. Thus, it is not appropriate to utilize single orthogonal wavelet directly in a WDMA-UWB system. So in this study, the method of designing pulse waveform for UWB wireless communication system, combined waveform, is proposed and the performance of this new method in WDMA-UWB system will be studied below.

The paper is organized as follows. In Section 2, the WDMA-UWB system model is introduced. Then in Section 3, the orthogonal wavelet definition is illustrated.  Section 4 analyzes the correlation property of single orthogonal wavelet and the combined waveform. In Section 5, simulation experiments that compare the performance of different combined waveform in both synchronous and asynchronous WDMA-UWB system are analyzed, followed by conclusions given in Section 6.

## 2. WDMA-UWB System Model

### 2.1. Signal Transmitting Model

Assume that there are *K* users in the WDMA-UWB system. Each transmitter employs binary phase-shift key (BPSK) modulation. At the transmitter *k*  (*k* = 1,2,…, *K*), orthogonal wavelets *w*_*k*_(*t*) with the duration *T*_*p*_ are used as the pulse waveforms, which are modulated by the BPSK symbols *b*_*k*_(*i*) ∈ {−1,+1}_*i*=1_ whose duration can be represented by *T*_*s*_. *M* bits of information signal transmitted by *k*th user can be described as follows:

(1) $$\begin{array}{c} x_{k}\left(t\right)=\overset{M}{\underset{i=1}{\sum }}b_{k}\left(i\right)w_{k}\left(t-iT_{s}-\tau_{k}\right),\; \end{array}$$

where *τ*_*k*_ denotes the random delay of the *k*th signal transmitted in the channel.

### 2.2. Signal Receiving Model

The traditional receiver of a WDMA-UWB system consists of a pulse demodulator and a matched filter corresponding to each user. The signal arriving at the matched filters can be described as follows:

(2) $$\begin{array}{c} r\left(t\right)=Ax\left(t-\tau \right)+n\left(t\right), \end{array}$$

where *A* denotes the channel gain, *x*(*t*) denotes the signal of users, *τ* denotes the random delay in the channel, and *n*(*t*) denotes the additive white Gaussian noise (AWGN) with the mean of zero and the unilateral power spectral density of *N*_0_.

**Figure 1:**
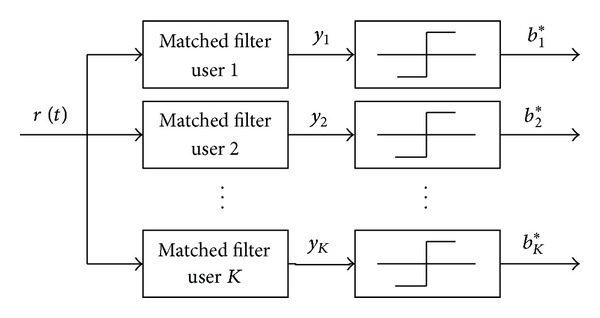
Matched filter part in the receiver.

The matched filter part of the receiver is shown in Figure 1.

Let vector **y** = [*y*_1_, *y*_2_,…,*y*_*K*_]^*T*^ denote the output of the group of matched filter and vector b* = [*b*_1_*, *b*_2_*,…,*b*_K_*]^*T*^ denote the output of sign detectors. So the output of the matched filters can be expressed as follows:

(3) $$\begin{array}{c} y=RAb+n,\\ b^{*}=\mathrm{sgn}\left(y\right), \end{array}$$

where the vector **b** = [*b*_1_, *b*_2_,…,*b*_*K*_]^*T*^ denotes the correct bits of each user, the vector **n** = [*n*_1_, *n*_2_,…,*n*_*K*_]^*T*^ denotes the output of the AWGN from each user's corresponding matched filter, **R** = (*r*_*ij*_)_*K*×*K*_ denotes the mutual correlation matrix of all users, and the element of matrix *r*_*ij*_ = *w*_*i*_(*t*)*w*_*j*_(*t*) and **A** = diag⁡(*A*_1_, *A*_2_,…, *A*_*K*_) in which diagonal element *A*_*k*_  (*k* ∈ [1, *K*], *k* ∈ *N*) represents the signal amplitude of the *k*th user.

## 3. Overview on Orthogonal Wavelets

### 3.1. Wavelet Definition

Wavelets consist of a group of functions that satisfy the following formula:

(4) $$\begin{array}{c} C_{\psi }=\overset{+\infty }{\underset{-\infty }{\int }}\frac{{\left|\Psi \left(\omega \right)\right|}^{2}}{\omega }d\omega <\infty ,\; \end{array}$$

where Ψ(*ω*) is the Fourier transform of *ψ*(*t*). *ψ*(*t*) is referred to as basic wavelet or mother wavelet. For arbitrary real number *a*, *b* ∈ *R*, *a* ≠ 0, *ψ*_*a*,*b*_(*t*) means that

(5) $$\begin{array}{c} \psi_{a,b}\left(t\right)={{\left|a\right|}^{-1/2}}^{}\psi \left(\frac{t-b}{a}\right). \end{array}$$

This kind of functions, such as *ψ*_*a*,*b*_(*t*) is called wavelets.

The constant of (4) limits *ψ*(*t*) to satisfy the following integral formulas:

(6) $$\begin{array}{c} \overset{+\infty }{\underset{-\infty }{\int }}\left|\psi \left(t\right)\right|dt<+\infty ,\\ \overset{+\infty }{\underset{-\infty }{\int }}\psi \left(t\right)dt=0. \end{array}$$

### 3.2. Orthogonal Wavelet Definition

An orthogonal wavelet is a function *ψ*(*t*)∈*L* ^2^(*R*) such that the double indexed set

(7) $$\begin{array}{c} {\left\{\psi_{k,j}\left(t\right)=2^{-k/2}\psi \left(2^{-k}t-j\right)\right\}}_{(k,j)\in Z\times Z} \end{array}$$

is an orthogonal basis of *L* ^2^(*R*).

According to the definition of orthogonality, orthogonal wavelet has the following property:

(8) $$\begin{array}{c} \langle \psi_{k,j}\left(t\right)\cdot \psi_{m,n}\left(t\right)\rangle =\delta \left(k-m,j-n\right), \end{array}$$

where *k*, *j*, *m*, and *n* are any integers and 〈·〉 denotes the inner product operation.

In this study, *ψ*(*t*) is an orthogonal wavelet. Kernel function is *ψ*_0,0_(*t*). According to (8), *ψ*_0,0_(*t*), *ψ*_0,1_(*t*), *ψ*_1,0_(*t*), *ψ*_1,1_(*t*), *ψ*_1,2_(*t*),… are mutually orthogonal.

## 4. Design of Pulse Waveform

### 4.1. Study on Correlation Property of Single Orthogonal Wavelet Function

#### 4.1.1. Synchronous Correlation Property

There are four common categories of orthogonal wavelets used in WDMA system, Daubechies wavelet, Symlets wavelet, Coiflets wavelet, and Meyer wavelet. In each category, one kind of wavelets is chosen to be studied through the simulation. In the following simulation, three wavelet functions *ψ*_0,0_(*t*), *ψ*_0,1_(*t*), and *ψ*_1,0_(*t*) are used as examples and compared their self-correlation properties and mutual correlation properties.

**Figure 2:**
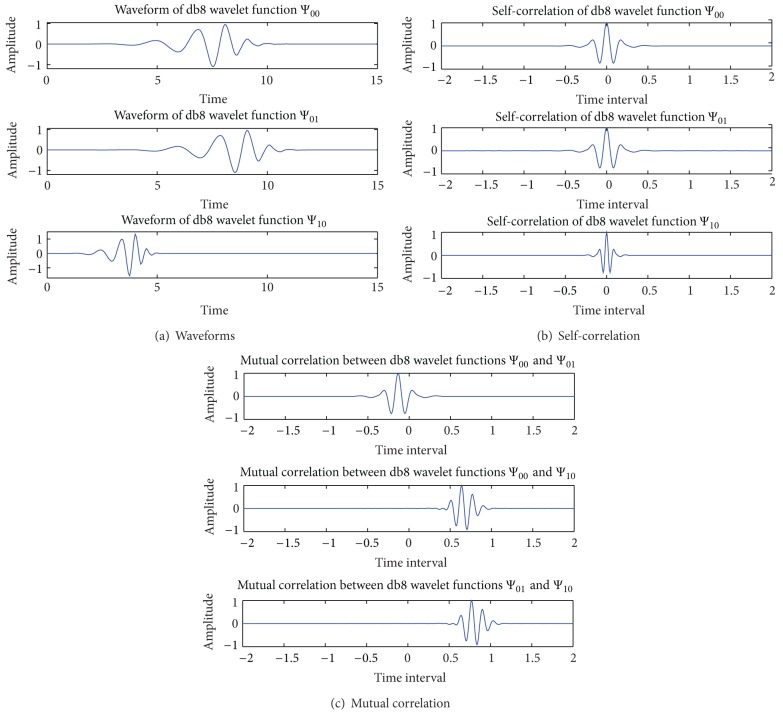
Waveform of db8 functions and their correlation property.

In the category of Daubechies, set db8 wavelet as an example. The waveforms, self-correlation properties, and mutual correlation properties of the three wavelet functions are shown in Figures 2(a), 2(b), and 2(c), respectively.

From Figure 2(b), it can be seen that the self-correlation values of these three wavelet functions are all one when the time interval is zero. At the same time interval in Figure 2(c), all of the values of mutual correlation are approximately zero.

Similar performances and the same conclusions can be obtained from the simulations of other three orthogonal wavelets, sym8 wavelet, coif5 wavelet, and Meyer wavelet.

In addition, the theoretical calculation values of mutual correlation of three wavelet functions in each category are shown in Table 1.

From Table 1, it can be obtained that all the values of mutual correlation are so small that they even can be regarded as zero. Comparing the four categories of orthogonal wavelets, the three mutual correlation values of db8 orthogonal wavelet are the smallest ones. All of these come to the conclusion that in the synchronous system, orthogonal wavelets are suitable to be used as the UWB pulse waveform in the WDMA system.

#### 4.1.2. Asynchronous Correlation Property

Synchronous system is an ideal condition. In the actual communication system in outer space, the signal transmitted by each user at the same time may not reach the receiver corresponding to each user at the same time. So there is great need to study the correlation property of single orthogonal wavelet in the asynchronous system.

In order to research the mutual correlation property of two wavelet functions in an asynchronous way, fix one wavelet function and translate the other wavelet function point by point. At each point, calculate the value of mutual correlation of the two functions. At last, compute the statistical mean, variance, and maximum of all the values of mutual correlation.

**Figure 3:**
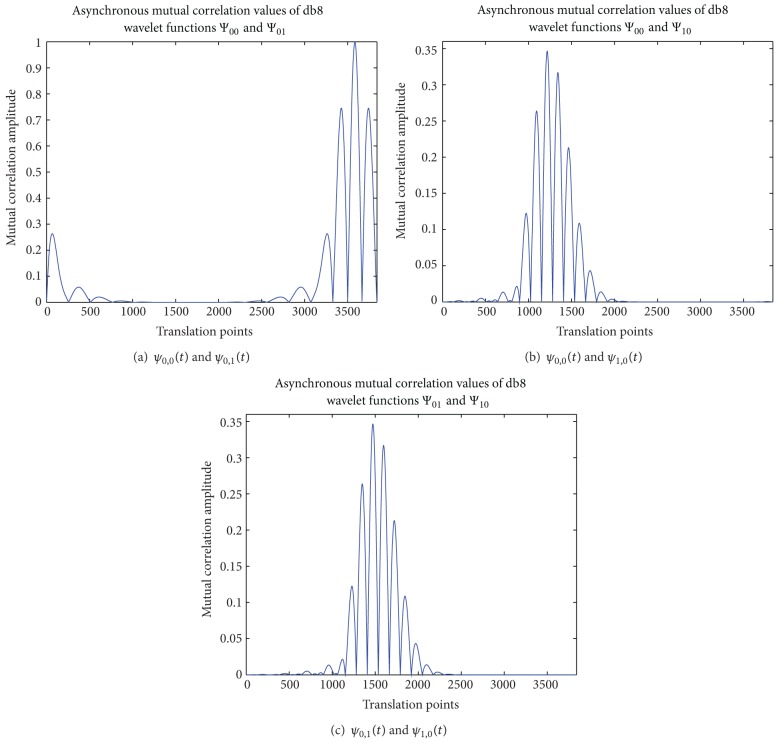
Asynchronous mutual correlation values of three db8 wavelet functions.

Take db8 orthogonal wavelet as an example. Fix *ψ*_0,0_(*t*) and translate *ψ*_0,1_(*t*) point by point. The value of mutual correlation changes with the translation point, which is shown in Figure 3(a). And similar figures about the asynchronous mutual correlation between *ψ*_0,0_(*t*) and *ψ*_1,0_(*t*) and between *ψ*_0,1_(*t*) and *ψ*_1,0_(*t*) are also shown in Figures 3(b) and 3(c), respectively.

As is known above, the value of self-correlation of each wavelet function is one. From Figure 3(a), it can be seen that the maximum of mutual correlation value is also one, which is not almost zero as expected. This result can explain that *ψ*_0,1_(*t*) is formed by the translation of *ψ*_0,0_(*t*) and these two wavelet functions can be completely the same at certain translation point. Thus, at this special point, the value of mutual correlation is equal to the value of self-correlation.

Comparing Figures 3(a) and 3(b), the values of mutual correlation are much smaller in Figure 3(b) than those in Figure 3(a), which is because *ψ*_1,0_(*t*) is formed by the compression of *ψ*_0,0_(*t*) and their shapes are not the same. So translating *ψ*_1,0_(*t*) point by point, the values of mutual correlation are much less than the value of self-correlation. Same explanation can also be used in Figure 3(c).

Similar waveforms and the same conclusions can be obtained from the simulations of other three orthogonal wavelets, sym8 wavelet, coif5 wavelet, and Meyer wavelet. The statistical mean, variance, and maximum of all the values of mutual correlation are shown in Tables 2, 3, 4, and 5, respectively.

The first statistical data, mean, is for the average value of mutual correlation. The second one, variance, is for the stability of mutual correlation following with the change of the translation points. And the last one, maximum, is for the maximum possible interference between users in WDMA-UWB system.

From Tables 2–5, it comes to two conclusions. The first one is that comparing four kinds of wavelets and considering the three aspects of statistics, the correlation property of Meyer wavelet is the best. The second one is that the statistics of *ψ*_0,0_(*t*) and *ψ*_1,0_(*t*) are much smaller than those of *ψ*_0,0_(*t*) and *ψ*_0,1_(*t*). In other words, the correlation property of two wavelet functions with different monocycles is much better than those with the same monocycle. To form pulse waveforms with good correlation property for WDMA-UWB system, it is advised to distribute wavelet functions with different monocycles to different users. And these two discoveries lay the foundation for designing combined waveform below.

### 4.2. Study on Correlation Property of Combined Waveform

#### 4.2.1. Synchronous Correlation Property

According to the research mentioned above, Meyer wavelet is chosen among the four categories as the foundation for designing combined waveform. In this study, there are three kinds of combination. To distinguish the differences between three kinds of combination, suppose there are two users.

1. Combination 1:
   user 1:  *ψ* ^(1)^(*t*) = *ψ*_0,0_(*t*) + *ψ*_0,1_(*t*),
   user 2:  *ψ* ^(2)^(*t*) = *ψ*_0,0_(*t*) − *ψ*_0,1_(*t*).
2. Combination 2:
   user 1:  *ψ* ^(1)^(*t*) = *ψ*_0,0_(*t*) + *ψ*_1,0_(*t*),
   user 2:  *ψ* ^(2)^(*t*) = *ψ*_0,1_(*t*) + *ψ*_1,1_(*t*).
3. Combination 3:
   user 1:  *ψ* ^(1)^(*t*) = *ψ*_0,0_(*t*) + *ψ*_0,1_(*t*),
   user 2:  *ψ* ^(2)^(*t*) = *ψ*_1,0_(*t*) + *ψ*_1,1_(*t*).

**Figure 4:**
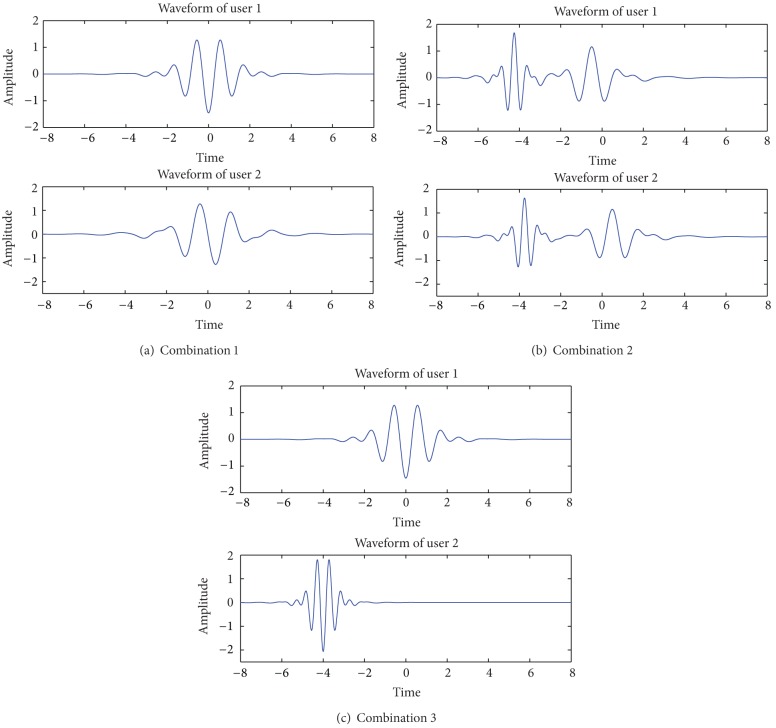
Waveforms of three combination modes.

The waveforms of three kinds of combination modes are shown in Figures 4(a), 4(b), and 4(c), respectively.

Take combination 1 for example, to prove the orthogonality between two users. Consider

(9) 〈ψ(1)(t)·ψ(2)(t)〉 =〈[ψ0,0(t)+ψ0,1(t)]·[ψ0,0(t)−ψ0,1(t)]〉 =〈ψ0,0(t)·ψ0,0(t)〉−〈ψ0,0(t)·ψ0,1(t)〉  +〈ψ0,1(t)·ψ0,0(t)〉−〈ψ0,1(t)·ψ0,1(t)〉 =〈ψ0,0(t)·ψ0,0(t)〉−〈ψ0,1(t)·ψ0,1(t)〉=0.

**Figure 5:**
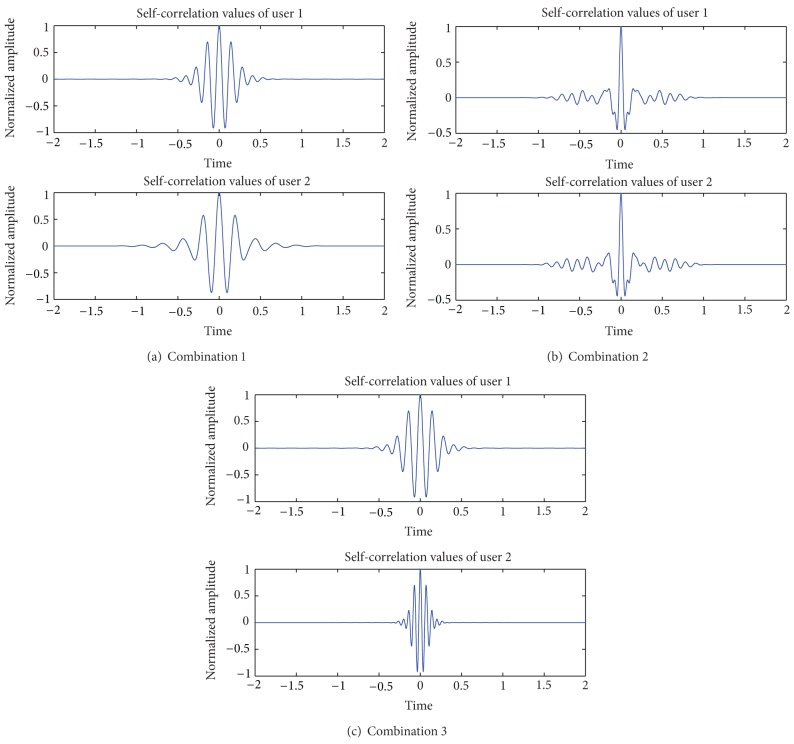
Self-correlation values of three combination modes.

In synchronous system, the correlation properties, especially the self-correlation values of the combined orthogonal wavelet and the theoretical value of mutual correlation between two users, of these three combinations, are arranged in Figures 5(a), 5(b), and 5(c) and Table 6.

From Table 6, the value of combination 1 is the smallest among the three and is even smaller than that of single wavelet function. Thus, combination 1 can be chosen as the combination mode to form pulse waveform for WDMA-UWB synchronous communication system.

#### 4.2.2. Asynchronous Correlation Property

**Figure 6:**
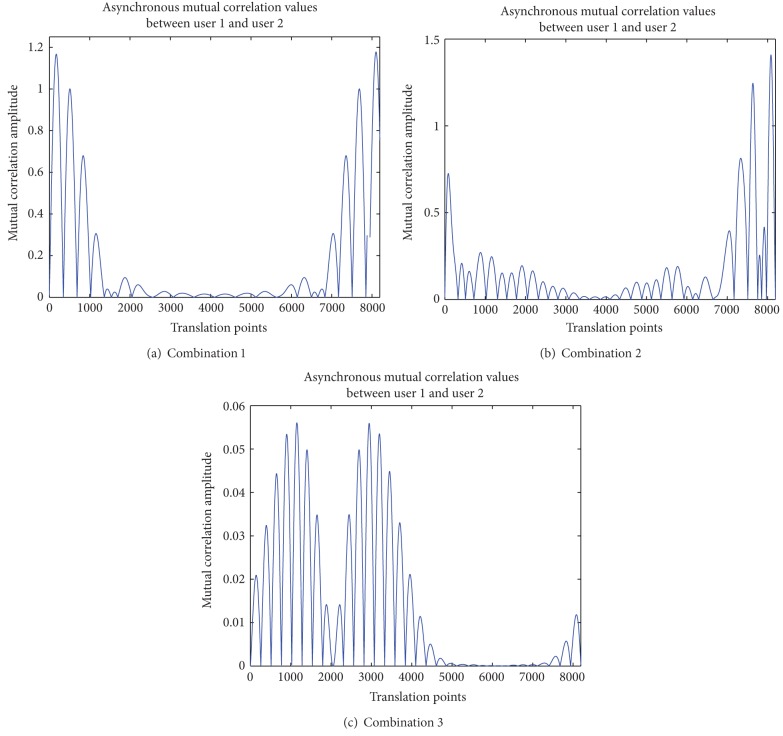
Asynchronous mutual correlation values of three combinations.

The method of studying the mutual correlation of combined waveform in an asynchronous way is the same with that of single wavelet function. Fix the waveform of user 1 and translate the waveform of user 2 point by point. The results of three combinations are shown in Figures 6(a), 6(b), and 6(c), respectively.

Additionally, the statistical mean, variance, and maximum of all the values of mutual correlation for three combination modes are shown in Table 7.

In the research of single wavelet function, the self-correlation value of single wavelet function is one. And in the simulation of combined waveform including two wavelet functions, the self-correlation value of one combined waveform is two. From Table 7, it is obviously to find that the maximum of mutual correlation for combinations 1 and 2 is greater than one. Take combination 2 as an example for explanation. The wavelet function *ψ*_0,0_(*t*) in user 1 is nearly the same with the function *ψ*_0,1_(*t*) in user 2. At certain translation point, the mutual correlation value of these two functions can be one, which is the self-correlation value of one wavelet function. Furthermore, at this special translation point, continue to calculate the mutual correlation of *ψ*_1,0_(*t*) in user 1 and *ψ*_1,1_(*t*) in user 2. The sum of two mutual correlation values must be greater than one.

From Table 7, comparing with the first two combination modes, the value of combination 3 is the smallest in all statistical data, mean, variance, and maximum. Therefore, combination 3 is the most suitable for combined waveform in WDMA-UWB asynchronous system.

## 5. Simulation and Discussion

### 5.1. Synchronous WDMA-UWB System

Suppose that it is a synchronous WDMA-UWB system and there are 10 users in this system. The waveform of each user is given below. Each user transmits 32 bits of information continuously each time and repeats transmitting for 8000 times. Then, calculate bit error rate (BER) with the signal noise ratio (SNR) from 0 dB to 10 dB.

1. Waveform of each user using combination 1:
   user 1:  *ψ* ^(1)^(*t*) = *ψ*_0,0_(*t*) + *ψ*_0,1_(*t*),
   user 2:  *ψ* ^(2)^(*t*) = *ψ*_0,0_(*t*) − *ψ*_0,1_(*t*),
   user 3:  *ψ* ^(3)^(*t*) = *ψ*_1,0_(*t*) + *ψ*_1,1_(*t*),
   user 4:  *ψ* ^(4)^(*t*) = *ψ*_1,0_(*t*) − *ψ*_1,1_(*t*),
   user 5:  *ψ* ^(5)^(*t*) = *ψ*_2,0_(*t*) + *ψ*_2,1_(*t*),
   user 6:  *ψ* ^(6)^(*t*) = *ψ*_2,0_(*t*) − *ψ*_2,1_(*t*),
   user 7:  *ψ* ^(7)^(*t*) = *ψ*_3,0_(*t*) + *ψ*_3,1_(*t*),
   user 8:  *ψ* ^(8)^(*t*) = *ψ*_3,0_(*t*) − *ψ*_3,1_(*t*),
   user 9:  *ψ* ^(9)^(*t*) = *ψ*_4,0_(*t*) + *ψ*_4,1_(*t*),
   user 10:  *ψ* ^(10)^(*t*) = *ψ*_4,0_(*t*) − *ψ*_4,1_(*t*).
2. Waveform of each user using combination 2:
   user 1:  *ψ* ^(1)^(*t*) = *ψ*_0,0_(*t*) + *ψ*_1,2_(*t*),
   user 2:  *ψ* ^(2)^(*t*) = *ψ*_0,1_(*t*) + *ψ*_1,3_(*t*),
   user 3:  *ψ* ^(3)^(*t*) = *ψ*_1,0_(*t*) + *ψ*_2,2_(*t*),
   user 4:  *ψ* ^(4)^(*t*) = *ψ*_1,1_(*t*) + *ψ*_2,3_(*t*),
   user 5:  *ψ* ^(5)^(*t*) = *ψ*_2,0_(*t*) + *ψ*_3,2_(*t*),
   user 6:  *ψ* ^(6)^(*t*) = *ψ*_2,1_(*t*) + *ψ*_3,3_(*t*),
   user 7:  *ψ* ^(7)^(*t*) = *ψ*_3,0_(*t*) + *ψ*_4,2_(*t*),
   user 8:  *ψ* ^(8)^(*t*) = *ψ*_3,1_(*t*) + *ψ*_4,3_(*t*),
   user 9:  *ψ* ^(9)^(*t*) = *ψ*_4,0_(*t*) + *ψ*_5,2_(*t*),
   user 10:  *ψ* ^(10)^(*t*) = *ψ*_4,1_(*t*) + *ψ*_5,3_(*t*).
3. Waveform of each user using combination 3:
   user 1:  *ψ* ^(1)^(*t*) = *ψ*_0,0_(*t*) + *ψ*_0,1_(*t*),
   user 2:  *ψ* ^(2)^(*t*) = *ψ*_1,0_(*t*) + *ψ*_1,1_(*t*),
   user 3:  *ψ* ^(3)^(*t*) = *ψ*_2,0_(*t*) + *ψ*_2,1_(*t*),
   user 4:  *ψ* ^(4)^(*t*) = *ψ*_3,0_(*t*) + *ψ*_3,1_(*t*),
   user 5:  *ψ* ^(5)^(*t*) = *ψ*_4,0_(*t*) + *ψ*_4,1_(*t*),
   user 6:  *ψ* ^(6)^(*t*) = *ψ*_5,0_(*t*) + *ψ*_5,1_(*t*),
   user 7:  *ψ* ^(7)^(*t*) = *ψ*_6,0_(*t*) + *ψ*_6,1_(*t*),
   user 8:  *ψ* ^(8)^(*t*) = *ψ*_7,0_(*t*) + *ψ*_7,1_(*t*),
   user 9:  *ψ* ^(9)^(*t*) = *ψ*_8,0_(*t*) + *ψ*_8,1_(*t*),
   user 10:  *ψ* ^(10)^(*t*) = *ψ*_9,0_(*t*) + *ψ*_9,1_(*t*).

**Figure 7:**
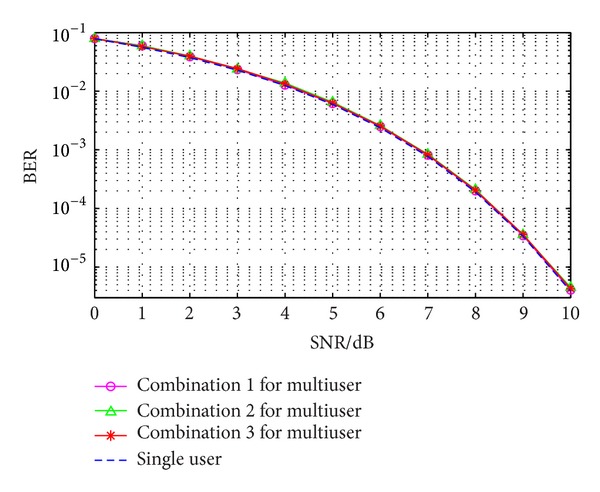
Performance of each combination mode in a synchronous system.

The performance of each combination mode in a synchronous WDMA-UWB system is shown in Figure 7.

From Figure 7, it can be seen that all the BER of three combination modes for multiuser are approximately the same with that of single user. It is hard to distinguish the difference of performance between these three combination modes. This performance of multiuser communication system is excellent for the reason that the synchronous correlation properties of all these three combination modes are very admirable. It can also be concluded that orthogonal wavelets are suitable for WDMA-UWB system as communication medium.

### 5.2. Asynchronous WDMA-UWB System

**Figure 8:**
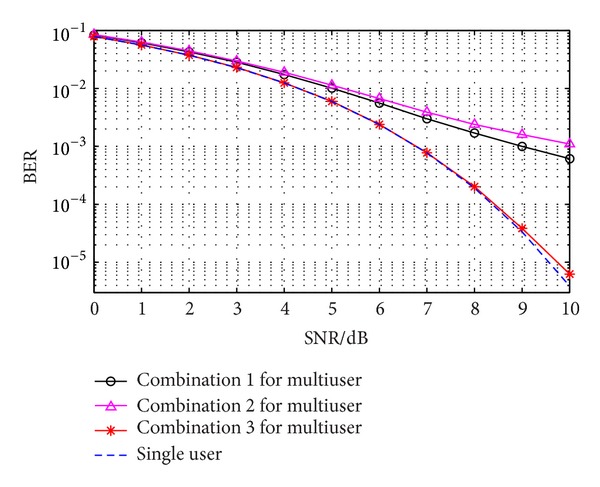
Performance of each combination mode in an asynchronous system.

With the same simulation parameters, research the BER of three combination modes for multiuser in an asynchronous WDMA-UWB system. The result and differences can be seen in Figure 8.

Seen from Figure 8, among three combination modes, the performance of combination 2 is the worst, while combination 3 is the best, which is the most close to the communication performance of single user. Demonstrated in Figure 5(c), the lowest BER of combination 3 is due to the best asynchronous correlation property among the three. However, the asynchronous correlation properties of combinations 1 and 2 are much worse than those of combination 3. And this can explain that the BER of first two combinations are much higher. Moreover, considering combination 2, the waveform of user 1 is almost the same as that of user 2, which can be easily confused in an asynchronous way. Thus, it can be explained why the performance of combination 2 is the worst.

## 6. Conclusions

The multiple access interference is the main reason that limits the UWB system capacity. In this study, a new multiple access scheme, WDMA based on the orthogonal wavelet function, is presented. After studying the correlation property of different categories of single wavelet functions, Meyer wavelet with the best correlation property is chosen to be the foundation for combined waveform. Each user is assigned to different combined orthogonal waveform. Proved by simulation, combined waveform is more suitable than single wavelet function to be a communication medium in WDMA system. Due to the excellent orthogonality, the BER of multiuser with combined waveforms is so close to that of single user in a synchronous system and even in an asynchronous system without multiuser detection after matched filters in the receivers, by using the third combination mode. In future work, multiuser detection technology will be studied with the purpose of reducing the BER of utilizing combinations 1 and 2 as UWB pulses in an asynchronous WDMA system.

## Figures and Tables

**Table 1:** Theoretical calculation values of mutual correlation.

Two wavelet functions	db8 wavelet value of mutual correlation	sym8 wavelet value of mutual correlation	coif5 wavelet value of mutual correlation	Meyer wavelet value of mutual correlation
*ψ*_0,0_(*t*) *ψ*_0,1_(*t*)	1.7497 × 10^−13^	4.3351 × 10^−14^	1.2654 × 10^−10^	2.1762 × 10^−16^
*ψ*_0,0_(*t*) *ψ*_1,0_(*t*)	7.7623 × 10^−7^	−5.5633 × 10^−5^	6.8283 × 10^−7^	−4.2253 × 10^−4^
*ψ*_0,1_(*t*) *ψ*_1,0_(*t*)	−8.7764 × 10^−8^	9.3128 × 10^−6^	−3.8248 × 10^−8^	5.8239 × 10^−4^

**Table 2:** Statistics of asynchronous mutual correlation values for db8 wavelet.

Statistics data	*ψ*_0,0_(*t*) and *ψ*_0,1_(*t*)	*ψ*_0,0_(*t*) and *ψ*_1,0_(*t*)	*ψ*_0,1_(*t*) and *ψ*_1,0_(*t*)
Mean	0.0927	0.0307	0.0307
Variance	0.0401	0.0050	0.0050
Maximum	1.0000	0.3467	0.3467

**Table 3:** Statistics of asynchronous mutual correlation values for sym8 wavelet.

Statistics data	*ψ*_0,0_(*t*) and *ψ*_0,1_(*t*)	*ψ*_0,0_(*t*) and *ψ*_1,0_(*t*)	*ψ*_0,1_(*t*) and *ψ*_1,0_(*t*)
Mean	0.0927	0.0303	0.0303
Variance	0.0401	0.0051	0.0051
Maximum	1.0000	0.3523	0.3523

**Table 4:** Statistics of asynchronous mutual correlation values for coif5 wavelet.

Statistics data	*ψ*_0,0_(*t*) and *ψ*_0,1_(*t*)	*ψ*_0,0_(*t*) and *ψ*_1,0_(*t*)	*ψ*_0,1_(*t*) and *ψ*_1,0_(*t*)
Mean	0.0507	0.0155	0.0155
Variance	0.0239	0.0024	0.0024
Maximum	1.0000	0.3074	0.3074

**Table 5:** Statistics of asynchronous mutual correlation values for Meyer wavelet.

Statistics data	*ψ*_0,0_(*t*) and *ψ*_0,1_(*t*)	*ψ*_0,0_(*t*) and *ψ*_1,0_(*t*)	*ψ*_0,1_(*t*) and *ψ*_1,0_(*t*)
Mean	0.1087	0.0281	0.0281
Variance	0.0427	0.0019	0.0019
Maximum	1.0000	0.1685	0.1685

**Table 6:** Theoretical values of mutual correlation of 3 kinds of combinations.

Combination mode	Value of mutual correlation between two users
Combination 1	−3.9328 × 10^−16^
Combination 2	−5.3033 × 10^−4^
Combination 3	−2.6554 × 10^−4^

**Table 7:** Statistics of asynchronous mutual correlation values of 3 combination modes.

Statistics data	Combination 1	Combination 2	Combination 3
Mean	0.1806	0.1582	0.0128
Variance	0.0887	0.0565	2.5405 × 10^−4^
Maximum	1.1673	1.4087	0.0560
